# Fine‐tuning of MPK6/3 phosphorylation by a lectin receptor‐like kinase LecRK‐VIII.2 regulates seed development

**DOI:** 10.1111/pbi.14135

**Published:** 2023-10-03

**Authors:** Wenjun Xiao, Shuai Hu, Keyao Yu, Yongliang Li, Ruqiong Cai, Daolong Xie, Haiwen Zhou, Ziming Guo, Shucan Liu, Xiaoxiao Zou, Shunxing Ye, Anping Guo, Ruifeng Yao, Hui Zhao, Xinhong Guo

**Affiliations:** ^1^ College of Biology Hunan University Changsha China; ^2^ Hainan Key Laboratory for Biosafety Monitoring and Molecular Breeding in Off‐Season Reproduction Regions, Institute of Tropical Bioscience and Biotechnology & San Ya Research Institute Chinese Academy of Tropical Agricultural Sciences Haikou China; ^3^ State Key Laboratory of Tropical Crop Breeding Chinese Academy of Tropical Agricultural Sciences Sanya China; ^4^ College of Bioscience and Biotechnology Hunan Agricultural University Changsha China

**Keywords:** LecRK‐VIII.2, MAPK cascade, seed development, *Arabidopsis*, rice

Receptor‐like kinases (RLKs) are of vital importance in transmembrane signalling, which coordinate plant growth, development, reproduction and environmental adaptation. MPK6/3, as the core components of MAPK cascade, are rapidly phosphorylated for activation when encountering endogenous signals or exogenous stimulus. Meanwhile, a relative low level of phosphorylated MPK6/3 (pMPK6/3) are maintained in healthy plants. The fluctuation of such constitutive pMPK6/3 dramatically reprograms plant growth and development (Zhang and Zhang, 2022), suggesting that the constitutive pMPK6/3 in a sophisticated range is strictly organized by various endogenous developmental signals. However, little is known about the regulatory mechanisms, primarily owing to our limited understanding about the specific RLKs for activating MAPK cascade. Here, we reveal that constitutive pMPK6/3 level in health plants is rigorously fine‐tuned by LecRK‐VIII.2 in both *Arabidopsis* and rice. LecRK‐VIII.2 regulates a range of MPK6/3‐related developmental phenotypes, including raisin‐like and burst seeds in *Arabidopsis*, and grain shape, filling, germination in rice.

We recently reported that *LecRK‐VIII.2* coordinated the trade‐off between seed size and quantity to determine seed yield in an MPK6‐dependant manner (Xiao *et al*., 2021). However, it remains vague whether LecRK‐VIII.2 is essential for the regulation of constitutive pMPK6/3 level. Thus, we detected the pMPK6 level in seedlings, inflorescence and seeds. The pMPK6/3 level was decreased in the tissues of *lecrk‐VIII.2* mutants, but markedly increased in the plants overexpressing *LecRK‐VIII.2* (Figure 1a), indicating that the constitutive pMPK6 level is positively regulated by LecRK‐VIII.2 at distinct developmental stages. Additionally, the yeast‐two‐hybrid assay showed that LecRK‐VIII.2 was unable to interact with MPK6/3 (Figure S1), suggesting that LecRK‐VIII.2 elevates pMPK6/3 level by an indirect way, possibly in a G protein/RLCKs‐MAPK cascade‐dependant manner.

**Figure 1:**
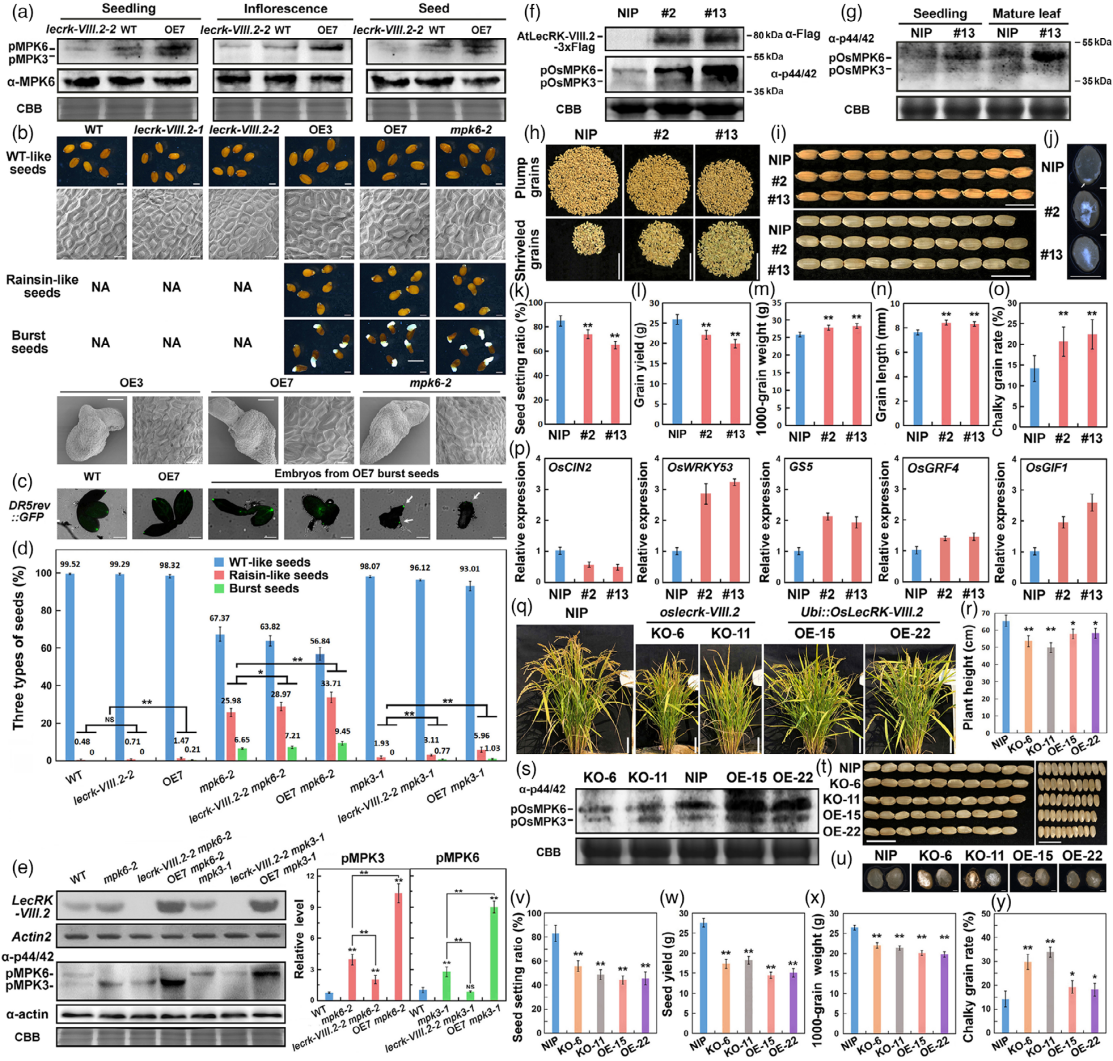
Functional characterization of LecRK‐VIII.2 on activation of MPK6/3 and regulation of seed development in *Arabidopsis* and rice. (a) pMPK6/3 level in 7 day seedlings, inflorescence and seeds. MAPK activation was analysed by α‐p44/42 antibody (CST, #9101). MPK6 level was detected using α‐MPK6 antibodies (Sigma, A7104). (b) The WT‐like seeds, raisin‐like seeds and/or burst seeds (bar = 250 μm). NA means the lines cannot produce the type of seeds. The three types of (bar = 100/20 μm). (c) The auxin activity reported by *DR5rev::GFP* during embryogenesis bar = 50 μm. (d) The percentage of the three types of seeds (*n* > 3000). (e) *AtLecRK‐VIII.2* transcription level (*Actin2* as internal control. Cat#19291, #10102, Yeasen) and pMPK6/3 level in developing seeds from the mutants analysed. Actin protein level (α‐actin, Abmart, M20009) and CBB staining serve as loading control. (f) AtLecRK‐VIII.2‐3 × Flag protein level (α‐Flag, Abmart, R20008) and pOsMPK6/3 level in young panicles from NIP, #2 and #13 plants. (g) pOsMPK6/3 level in 15 days seedlings and mature leaves. (h–j) Plump and shrivelled grains per plant (bar = 5 cm, h), mature paddy rice grains and brown rice grains (bar = 1 cm, i) and chalky grains (bar = 250 μm, j). (k–o) Seed setting ratio (*n* = 10, k), grain yield (*n* = 10, l), 1000‐grain weight (m), grain length (*n* > 300, n) and chalky grain rate (*n* > 300, o). (p) Transcription level of *OsCIN2*, *OsWRKY53*, *GS5*, *OsGRF4* and *OsGIF1* (*OsUBQ5* as internal control. Cat#19291, #11201, Yeasen). (q, r) Mature plants of NIP, KO‐6, KO‐11, OE‐15 and OE‐22 (bar = 10 cm, q), plant height (*n* = 10, r). (s) pOsMPK6/3 level in 15 days seedlings. (t–y) Brown rice grains (bar = 1 cm, t) and chalky grains (bar = 1 mm, u), seed setting ratio (*n* = 10, v), grain yield (*n* = 10, w), 1000‐grain weight (x) and chalky grain rate (*n* > 300, k). Values are means ± SE (*n* = three bio replicates, e and p). *P < 0.05, **P < 0.01. NS no significance (Student's *t*‐test).

The observation exhibited that *lecrk‐VIII.2* mutants developed small seeds with decreased size and number of epidermal cells, while the OE lines formed large seeds with bigger and more epidermal cells than WT plants (Figure 1b; Figure S2a,b). Besides regulation on seed size, a few raisin‐like and burst seeds, a well‐known MPK6‐related phenotype, were observed in the OE lines, but not in *lecrk‐VIII.2* mutants (Figure 1b), indicating the functional redundancy among LecRK‐VIII.2 and its homologous genes during seed development, the burst seeds from OE lines and *mpk6‐2* mutant produced the epidermal cells with abnormal morphology, which lacked convex structure at cell edges (Figure 1b). The auxin activity reported by *DR5rec::GFP* was significantly disrupted in the embryo of defective OE7 seeds, which consistent with the developmental defects and the *DR5rev:GFP* patterns observed in seeds of MAPK cascade mutants, *mpk6*, *mpk3 mpk6/+*, *mkk4 mkk5* and *yda*. These results together suggest that the abnormal seed development caused by *LecRK‐VIII.2* overexpression is similar with that in the MAPK mutants. Given that both gain and loss of function of YDA, an MAPK cascade component activating MPK6/3, cause a range of abnormalities in embryo development (Zhang and Zhang, 2022), it is possible that both lower or higher pMPK6/3 level would disrupt the developmental balance between embryo and seed coat.

To further investigate the genetic and biochemical association between LecRK‐VIII.2 and MPK6/3, we generated *lecrk‐VIII.2 mpk6‐2*, OE7 *mpk6‐2*, *lecrk‐VIII.2 mpk3‐1* and OE7 *mpk3‐1* mutants (Figure 1d,e). *lecrk‐VIII.2 mpk6‐2* plants produced more raisin‐like seeds and burst seeds than *mpk6‐2* mutant. Similarly, *lecrk‐VIII.2 mpk3‐1* plants developed more burst seeds, though *mpk3‐1* formed no seeds with burst‐out embryo. Consistently, the abundance of pMPK3 and pMPK6 were reduced in *lecrk‐VIII.2 mpk6‐2* and *lecrk‐VIII.2 mpk3‐1* compared to *mpk6‐2* and *mpk3‐1*, respectively, indicating that the further reduction in pMPK6/3 level by *lecrk‐VIII.2* mutation in *mpk6‐2* and *mpk3‐1* background led to more severe imbalance between seed coat and embryo development. Meanwhile, OE7 *mpk6‐2* plants showed the highest ratio of raisin‐like seed and burst seed, and OE7 *mpk3‐1* plants likewise formed more raisin‐like and burst seeds when compared to OE7 and *mpk3‐1*, though the pMPK3 and pMPK6 level were significantly increased in OE7 *mpk6‐2* and OE7 *mpk3‐1* seeds. Given that the increased level of pMPK3 or pMPK6 by overexpressing *LecRK‐VIII.2* could not rescue phenotype of seed developmental defects caused by *mpk6* or *mpk3* mutation (Figure 1d,e), it is possible that MPK6 and MPK3 have different specific substrates that could allow the LecRK‐VIII.2‐mediated signalling to branch out to coordinate the seed coat and embryo development. Additionally, seedlings from raisin‐like seeds and burst seeds produced by OE7, *mpk6‐2*, OE7 *mpk6‐2* and OE7 *mpk3‐1* plants, developed asymmetric dicotyledons, monocotyledon and even short roots or no roots (Figure S2c,d), which also found in *mpk6* mutant. Together, these results indicate that LecRK‐VIII.2 works upstream of MPK6 and MPK3 to regulate seed development by fine‐tuning the level of both pMPK6/3 and unphosphorylated‐MPK6/3 in a sophisticated status.

MAPK cascades are highly conserved signalling pathways in *Arabidopsis* and rice (Ren *et al.*, 2023). We generated the transgenic lines *35S::AtLecRK‐VIII.2‐3* × *Flag* Nipponbare (NIP) (Table S1). The OE plants exhibited remarkably increased level of pOsMPK6/3 in young grains. Seedlings and mature leaves compared to NIP plants. Indicates that AtLecRK‐VIII.2 plays a conserved role in constitutively activating pOsMPK6/3.

The *AtLecRK‐VIII.2* OE lines showed dwarf plant type (Figure S3d), and produced grains with increased length and width, but reduced seed setting ratio, leading to decreased grain yield when compared to NIP plant (Figure 1h,i,k–n; Figure S3c–i). Meanwhile, the OE lines developed more chalky grains, and the chalkiness was mainly located in the heart of grain (Figure 1j,o; Figure S3j). The OE grains also showed strikingly inhibited α‐amylase activity in the starch board test (Figure S3k,l), and displayed decreased germination speed and rate than NIP grains (Figure S3m). Consistently, *OsCIN2*, a cell‐wall invertase gene regulating chalkiness, was significantly inhibited in the OE lines (Figure 1p). Together, these findings demonstrate that overexpression of *AtLecRK‐VIII.2* could increase pOsMPK6 to break the trade‐off between grain size and quantity to determine yield and produce more chalky grains with lower germination activity.

MPK6/3 are critical components for brassinolide (BR) signalling activation (Ren *et al.*, 2023; Zhang and Zhang, 2022). The expression of *OsWRKY53*, activated by pOsMPK6 to promote BR signalling and grain size, was elevated in the *AtLecRK‐VIII.2* OE lines (Figure 1p). Other BR signalling‐related genes downstream of MAPK cascade for grain size promotion, such as *GS5*, *OsGRF4* (*Growth Regulating Factor 4*, *GS2*/*GL2*) and *OsGIF1* (*GRF interacting factor 1*), were also significantly up‐regulated in the OE lines (Figure 1p), implying that LecRK‐VIII.2 may be a novel RLK involving in BR signalling to regulate grain development.

We generated knock‐out and overexpression lines of *OsLecRK‐VIII.2* (Table S1; Figure S4a–c; Figure 1q,r). The *oslecrk‐VIII.2* mutants showed slightly reduced pOsMPK6/3 level, while the *OsLecRK‐VIII.2* overexpression lines harboured significantly increased pOsMPK6/3 abundance, compared to NIP plants (Figure 1s). The *oslecrk‐VIII.2* mutants produced smaller grains with decreased 1000‐grain weight (Figure 1t), consistent with our previous study that *AtLecRK‐VIII.2* T‐DNA mutants formed small seeds (Xiao *et al*., 2021). Meanwhile, the *OsLecRK‐VIII.2* OE lines exhibited reduced plant height, seed setting ratio, seed yield and seed germination ratio, as well as increased chalkiness (Figure 1q,r,t–y; Figure S4d–f), similar with the observation in *AtLecRK‐VIII.2* OE lines. These results together support that *OsLecRK‐VIII.2* acts as a conserved RLK to constitutively activate OsMPK6/3 and regulate grain development.

Unexpectedly, the *OsLecRK‐VIII.2* OE lines developed smaller grains than NIP plants (Figure 1t,x; Figure S4d), although they harboured higher pOsMPK6/3 level (Figure 1s). Given that both overexpression and suppression of rice G‐protein β subunit RGB1, a scaffold associating RLK and MAPK cascade components, produce short grains (Ren *et al.*, 2023), it is possible that OsLecRK‐VIII.2 interacts with RGB1 or other G‐protein subunits to modulate grain size. Alternatively, excessively constitutive activation of OsMPK6/3 might break the balance between growth and defence, leading to the inhibited growth and developmental defects. The potential roles of OsLecRK‐VIII.2 in stress and defence responses would be worthy to explore in future studies.

In conclusion, we identify LecRK‐VIII.2 as a conserved RLK regulating seed development in both *Arabidopsis* and rice (Figure S5). This study could expand our understanding about the molecular mechanisms underlying how monocots and dicots constitutively fine‐tune pMPK6/3 level to determine seed development. Furthermore, it would be interesting to investigate the missing conserved ligand(s), which may act as a crucial endogenous regulator/hormone for plant developmental remodelling.

## Conflict of interest statement

The authors declare no conflict of interest.

## Authors' contributions

X.H.G., H.Z., R.F.Y., W.J.X., S.H. and A.P.G. conceived the research. W.J.X., S.H., K.Y.Y., R.Q.C., D.L.X, H.W.Z., Z.M.G. Y.L.L., S.C.L., X.X.Z. and S.X.Y. performed the experiments. X.H.G., H.Z., Y.F.Y., W.J.X. and S.H. analysed the data. W.J.X. and S.H. drafted the manuscript.

## Supporting information


**Figure S1 ‐ S5** Supplementary Figures.
**Table S1** Primers used in this work.
