# Supplementary material for: Fine‐tuning of MPK6/3 phosphorylation by a lectin receptor‐like kinase LecRK‐VIII.2 regulates seed development
Source: Plant Biotechnol J. 2023 Oct 3;21(12):2414–6. doi: 10.1111/pbi.14135 (PMC10651140; doi:10.1111/pbi.14135)
Supplement: Supplementary file 1 — Figure S1 ‐ S5 Supplementary Figures. Table S1 Primers used in this work. [file PBI-21-2414-s001.docx]

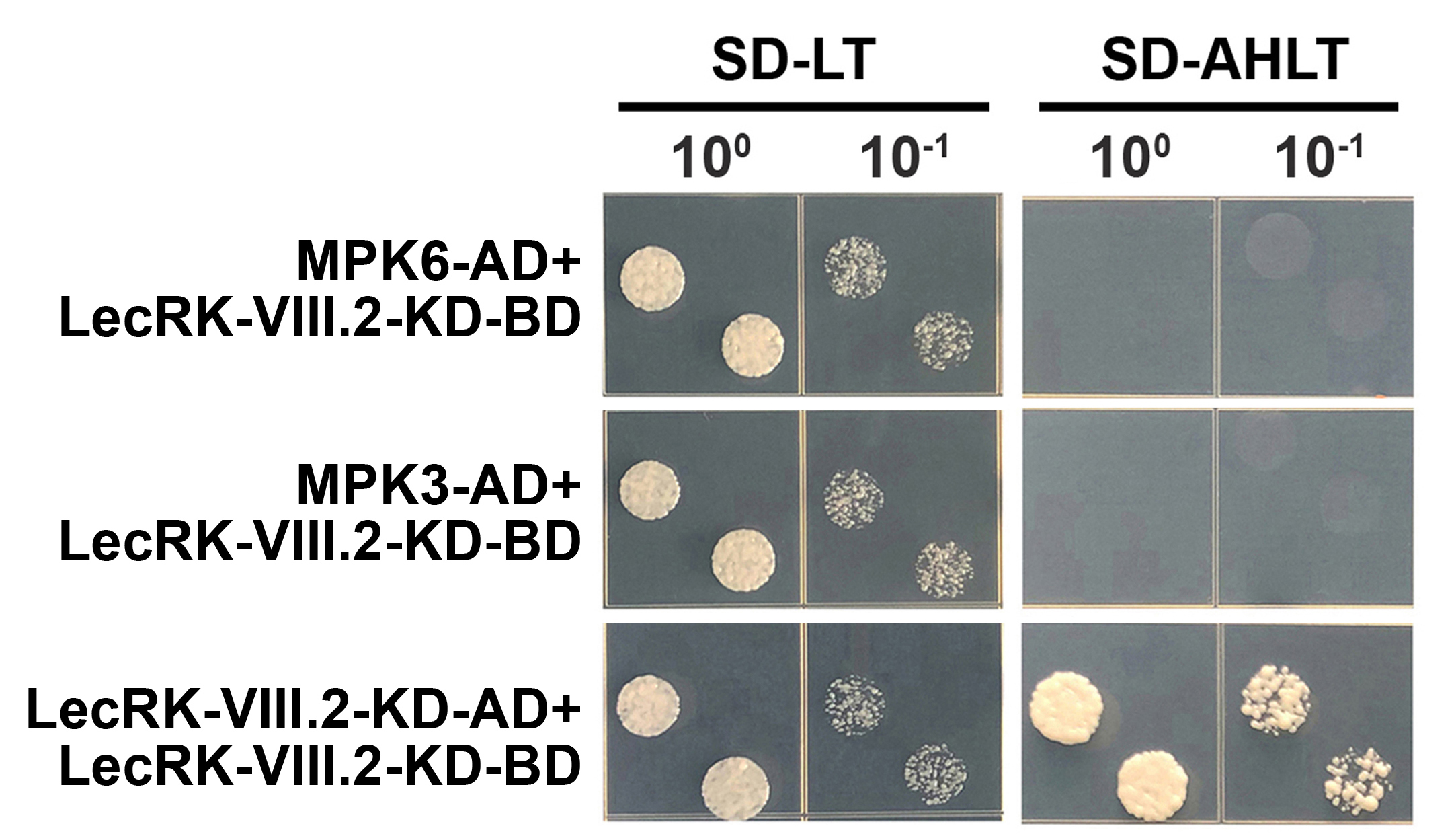


**Figure S1**. AtLecRK-VIII.2 is unable to interact with AtMPK6 and AtMPK3.

AtLecRK-VIII.2 can form homodimer (positive control). KD, kinase domain. Full-length CDS of AtMPK6/3, and the CDS of AtLecRK-VIII.2-KD were cloned into pGADT7 (AD) and pGBKT7 (BD), respectively. Yeast transformation was completed following the manufacturer's instructions (Clontech) and cultured on SD/-Leu-Trp or SD/-Ade-His-Leu-Trp medium at 30℃ for 5d. Primers used are given in Table S1.


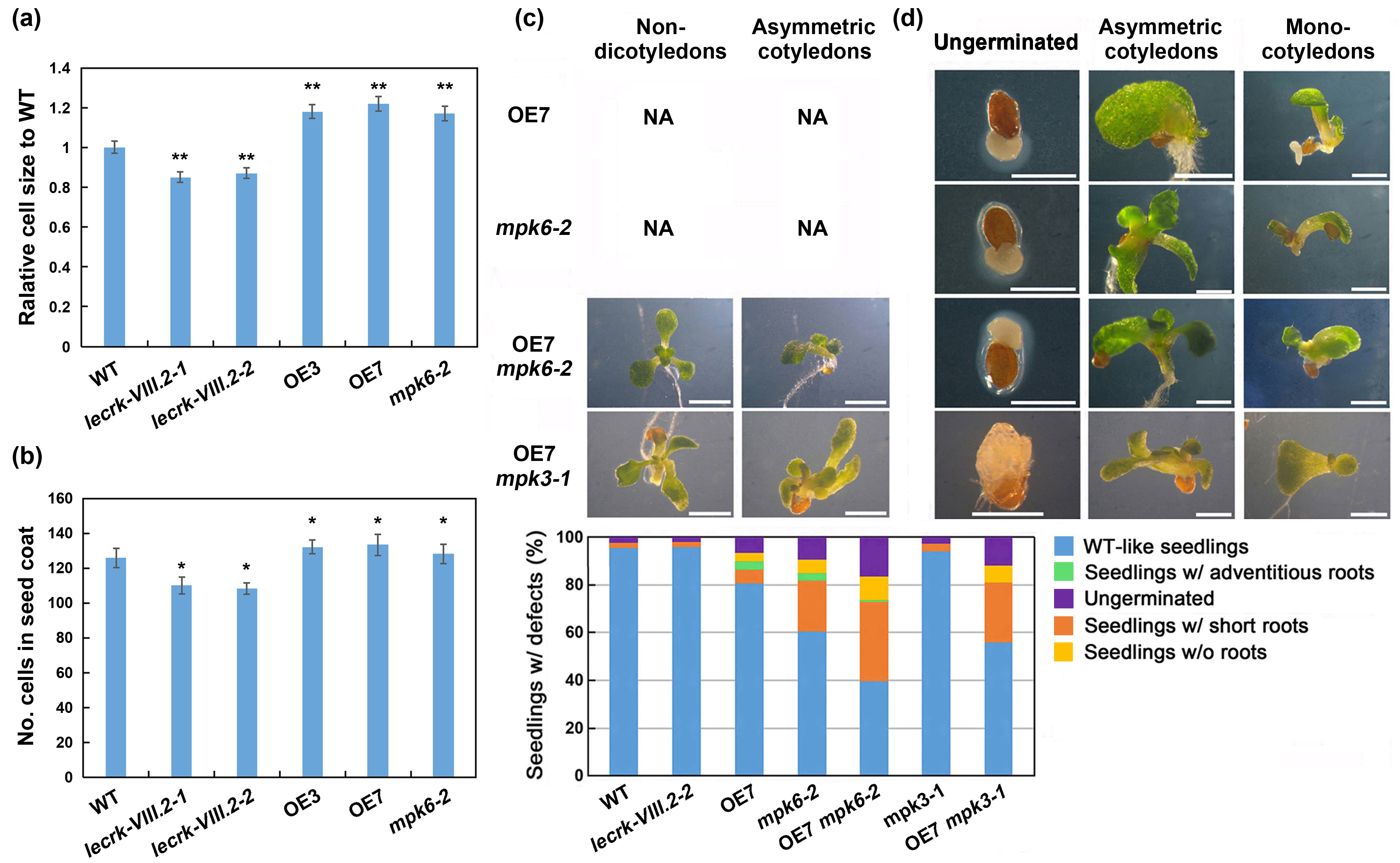


**Figure S2** LecRK-VIII.2-MPK3/6 module regulates cell expansion and proliferation in seed coat, and early development of seedlings.

(a) Relative cell size of *lecrk-VIII.2-1*, *lecrk-VIII.2-2*, OE3, OE7 and *mpk6-2* to WT. Values are means±SE (n>120 cells) relative to the WT value that is set at 1.

(b) The No. of cells in seed coat of WT, *lecrk-VIII.2-1*, *lecrk-VIII.2-2*, OE3, OE7 and *mpk6-2* (n>120 seeds).

(c,d) The seedlings from raisin-like seeds (N/A means the lines can not produce the type of seedlings, bar=3 mm, c), and the burst seeds and the seedlings germinated from OE7, *mpk6-2*, OE7 *mpk6-2* and OE7 *mpk3-1* (bar=1 mm, d). Percentage of seedlings with or without the indicated defects (n>100). *P < 0.05, **P < 0.01 compared with WT. (Student's *t* test).


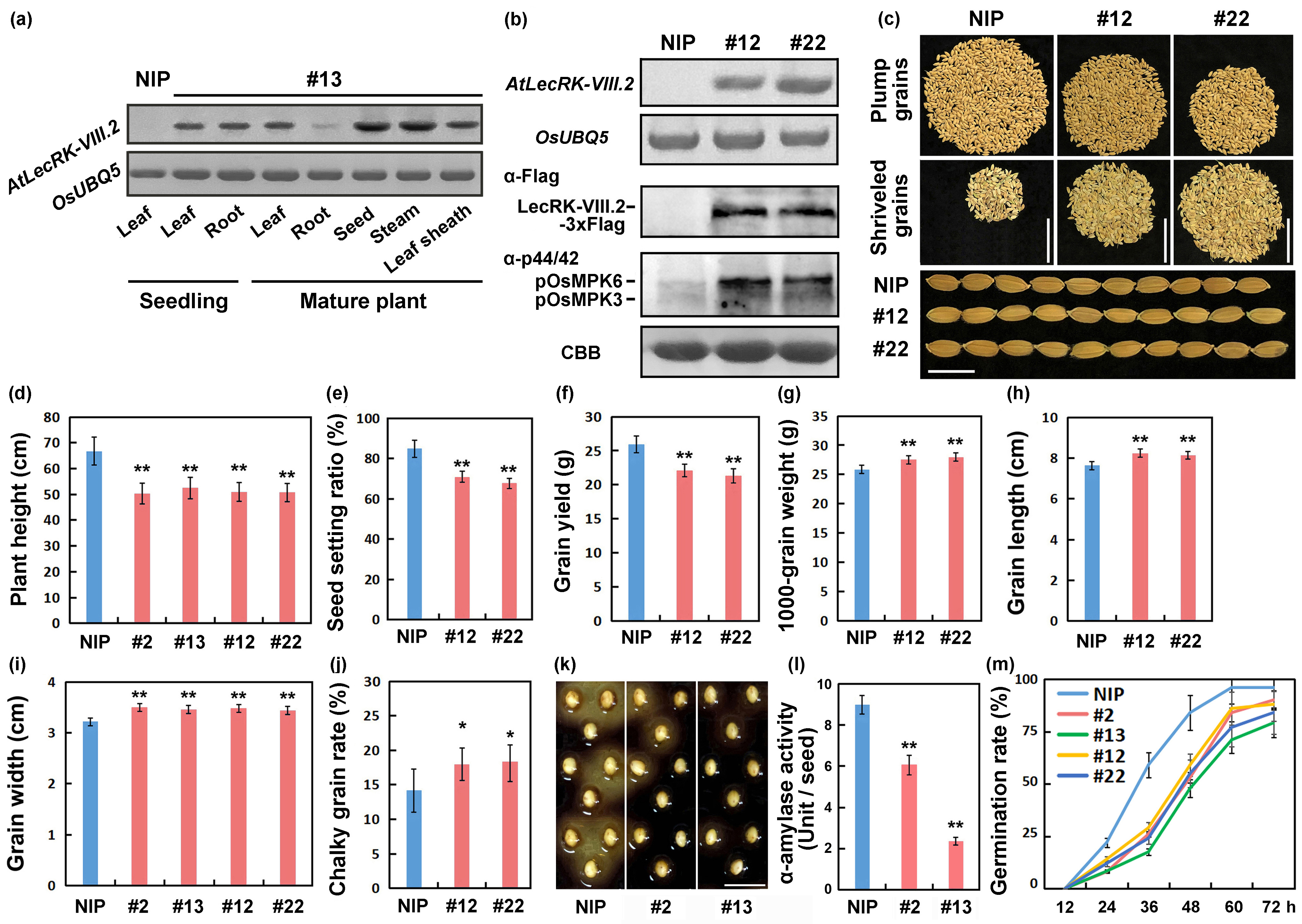


**Figure S3** The *35S::AtLecRK-VIII.2-3×Flag* transgenic rice plants display comparable phenotypes during plant growth, grain development and germination.

1. *AtLecRK-VIII.2* expression in the different parts of the #13 line. *OsUBQ5* acts as internal control.
2. *AtLecRK-VIII.2* expression level, AtLecRK-VIII.2-3×Flag protein level and pOsMPK6/3 level in young panicles of NIP, #12 and #22 plants. *OsUBQ5* and CBB staining work act as control.
3. The plump and shriveled grains per plant (bar=5cm), and mature paddy rice grains from NIP, #12 and #22 lines, bar=1 cm.

(d-j) Plant height (n=10, d), seed setting ratio (n=10, e), grain yield (n=10, f), 1000-grain weight (n=10, g), grain length (n>300, h), grain width (n>300, i), chalky grain rate (n>300, j) of NIP, #2, #13, #12 and #22 lines.

(k,l) The starch board test of seeds. The colourless halos produced by the half-seeds without embryos indicates α-amylase activity of the plants (bar=10 mm, n=27, k). Determination of α-amylase activity (Units/seeds) in the grains of NIP, #2 and #13 plants (n=6, l)

(m) Seed germination rate (n>100, k). *P < 0.05, **P < 0.01 compared with NIP (Student's *t* test).


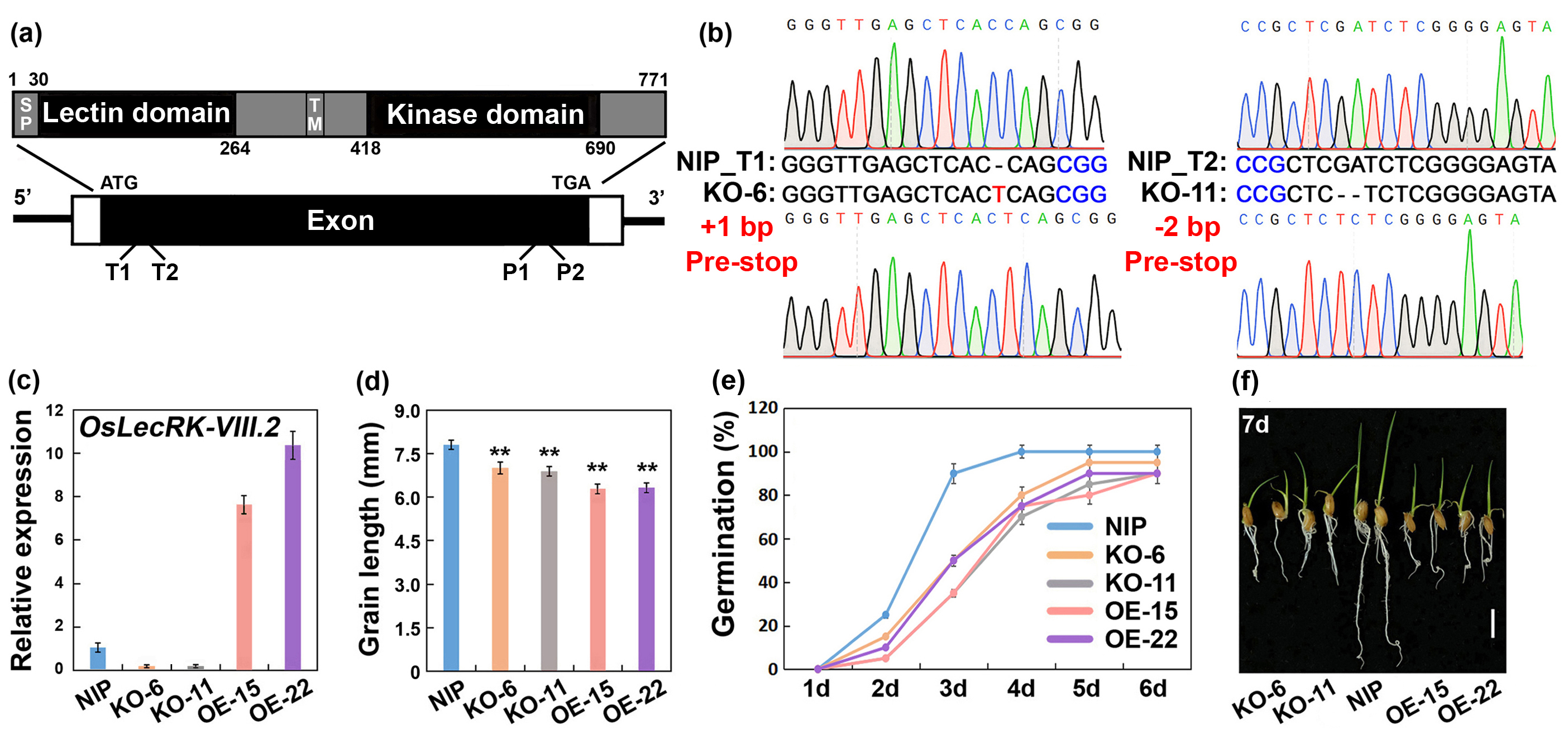


**Figure S4** *OsLecRK-VIII.2* regulates rice growth and development.

(a) The gene structure and protein domains of rice OsLecRK-VIII.2. The OsLecRK-VIII.2 protein contains signal peptide (SP), Lectin domain, transmembrane (TM) and kinase domain. T1 and T2 show the location of specific gRNA for gene-editing *OsLecRK-VIII.2*. P1 and P2 indicate the primers of OsLecRK-VIII.2-qF/R.

(b) Mutations in the gene-edited lines of KO-6 and KO-11.

(c) *OsLecRK-VIII.2* expression level in young panicles of NIP, KO-6, KO-11, OE-15 and OE-22. The primers (OsLecRK-VIII.2-qF/R) are showed in Table S1. Values are means±SE (n=three bio-replicates) relative to the NIP value that is set at 1.

(d-f) Grain length (n>300, d), seed germination rate (n=60 seeds, e) and 7d seedlings of NIP, KO-6, KO-11, OE-15 and OE-22 (bar=1 cm, f). **P < 0.01 compared with NIP, (Student's *t* test).


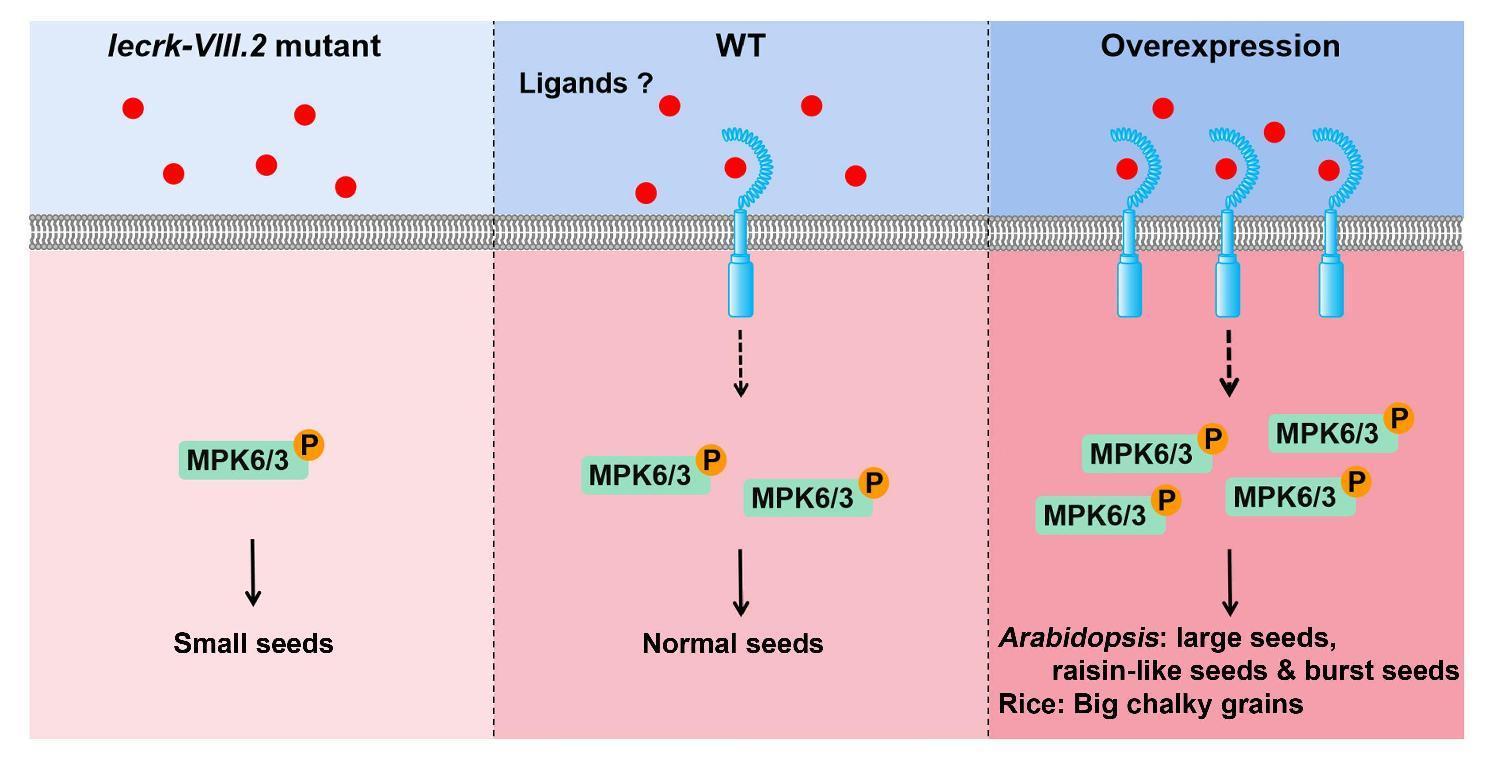


**Figure S5** A framework of seed development regulated by LecRK-VIII.2-MPK3/6 module in *Arabidopsis* and rice. In the presence of unknown ligand(s) as developmental signal, LecRK-VIII.2 controls seed development by fine-tuning the phosphorylation level of MPK6 and MPK3. *lecrk-VIII.2* mutants with decreased pMPK6/3 develop small seeds than WT plants. The *Arabidopsis* overexpressing *AtLecRK-VIII.2* activates MPK6/3 to produce large seeds and a few raisin-like seeds and burst seeds. The transgenic rice overexpressing *AtLecRK-VIII.2* forms big grains with increased chalkiness.

**Table S1. Primers used in this work.**

| pROK2-T | GGAACCACCATCAAACAGGA |
| --- | --- |
| *lecrk-VIII.2-2*-TF | GACCAATTTCCAAACCCTTTC |
| *lecrk-VIII.2-2*-TR | CTGCTTTGGCTTATTTGCATC |
| At8.2KD-BD-F | GTACCAGATTACGCTCATATGCCATCGTCCTCTTGCCGTAA |
| At8.2KD-BD-R | TCAGAGGAGGACCTGCATATGCCATCGTCCTCTTGCCGTAA |
| AtMPK3-AD-F | GCCATGGAGGCCAGTGAATTCATGAACACCGGCGGTGG |
| AtMPK3-AD-R | ATGCCCACCCGGGTGGAATTCACCGTATGTTGGATTGAGTGCT |
| AtMPK6-AD-F | GCCATGGAGGCCAGTGAATTCATGGACGGTGGTTCAGGTC |
| AtMPK6-AD-R | ATGCCCACCCGGGTGGAATTCTTGCTGATATTCTGGATTGAAAGCA |
| *LecRK-VIII.2*-qF | GCTACGGAGCCGTTGTTTTA |
| *LecRK-VIII.2*-qR | CCACCATCATAACCCGACTC |
| *Actin2*-qF | CACTGTGCCAATCTACGAGGGT |
| *Actin2*-qR | CACAAACGAGGGCTGGAACAAG |
| At8.2-1300nFLAG-XbaI-F | GAGAACACGGGGGACTCTAGAATGCCACCACCCATGGCGC |
| At8.2-1300nFLAG-XbaI-R | ATCCTTGTAATCCATTCTAGATCACCGGGCGGCGTCG |
| *hpt557*-F | ACACTACATGGCGTGATTTCAT |
| *hpt557*-R | TCCACTATCGGCGAGTACTTCT |
| Os8.2-1300n-F | GAGAACACGGGGGACTCTAGAATGCCACCACCCATGGCGC |
| Os8.2-1300n-R | ATCCTTGTAATCCATTCTAGATCACCGGGCGGCGTCG |
| *OsLecRK-VIII.2*-qF | CAGATGCTGGGCGGAGAG |
| *OsLecRK-VIII.2*-qF | TTGTAGTCGGACACGCTGTC |
| *OsUBQ5*-qF GCAGAAGCACAAGCACAAGAAG  OsUBQ5-R GCCTGCTGGTTGTAGACGTAGG | GCAGAAGCACAAGCACAAGAAG |
| *OsUBQ5*-qR | GCCTGCTGGTTGTAGACGTAGG |
| *OsCIN2*-qF | GACAGGTCGGTTGTTGAGAGCT |
| *OsCIN2*-qR | AGGCTCCATTCATCATGACCGG |
| *OsWRKY53*-qF | GTCCTCCTCAGCTCCTCCCATA |
| *OsWRKY53*-qR | TTGAAGGAAGGGAAAGAGGCCG |
| *GS5*-qF | TGTCTTCTCCTCCTCCTGTGCT |
| *GS5*-qR | GCGCCTCGAAGAACCAGTAGAA |
| *OsGIF1*-qF | CTGATGCAGATGAACCAGGGCA |
| *OsGIF1*-qR | GCGGCGAGGTACATGAGATTGT |
